# Impedance Spectroscopy as a Novel Approach to Probe the Phase Transition and Microstructures Existing in CS:PEO Based Blend Electrolytes

**DOI:** 10.1038/s41598-018-32662-1

**Published:** 2018-09-25

**Authors:** Shujahadeen B. Aziz, M. G. Faraj, Omed Gh. Abdullah

**Affiliations:** 1 Prof. Hameed’s Advanced Polymeric Materials Research Laboratory, Department of Physics, College of Science, University of Sulaimani, Sulaimani, Kurdistan Regional Government Iraq; 2 Komar Research Center (KRC), Komar University of Science and Technology, Sulaimani, 46001 Kurdistan Regional Government Iraq; 3 Department of Physics, Faculty of Science and Health, Koya University, University Park, Koysinjaq, Kurdistan Regional Government Iraq

## Abstract

In this work the role of phase transition of PEO from crystalline to amorphous phases on DC conductivity enhancement in chitosan-based polymer electrolyte was discussed. Silver ion-conducting polymer electrolytes based on chitosan (CS) incorporated with silver nitrate (AgNt) is prepared via solution cast technique. Various amounts of polyethylene oxide (PEO) are added to the CS:AgNt system to prepare blend polymer electrolytes. Ultraviolet-visible (UV-vis) spectrophotometry is used to confirm that the blended samples containing AgNt salt exhibit a broad absorption peak. From optical micrograph images it is apparent that small white specs appear on the surface of the samples. The SEM results clearly show the aggregated silver nanoparticles. The enlargement of the crystalline area was observed from the morphological emergence and impedance plots. The phase separation in SEM images was observed at high PEO concentration. The XRD consequences support the morphological manifestation. In this study a new approach is offered to explore the microstructures existing in the blend electrolytes. The width of the semicircle linked to crystalline phase in impedance spectra was found to be increased with the increase of PEO concentration. A slow increase of DC conductivity was observed at low temperatures while above 333 K an immediate change in DC conductivity was obtained. The rapid rise of DC conductivity at high temperatures is correlated with the DSC results and impedance studies at high temperatures.

## Introduction

Solid polymer electrolytes (SPEs) have emerged as a new class of electrolyte materials for the replacement of the conventional organic sol–gel electrolyte. This is due to their long life, safety, processability, flexibility, and both electrochemical and dimensional stabilities^1^. They are promising materials since they tend to eliminate other problems of harmful gas production and corrosive solvent leakage along with their wider applications in electrochemical devices, fuel cells and electrochromic windows^2^. Polymer blending is a convenient method to create novel polymeric materials that are able to yield with the superior property profiles as compared to those of the individual components. This method is usually far less costly and time-consuming for the production of polymeric materials with new properties^3^. The polymer blending is also known to be one of the most promising and possible ways to enhance the ionic conductivity of polymeric electrolyte membranes. However, a high degree of polymeric blending can result in poor mechanical properties in SPEs^4^. It is well reported that polymer electrolytes with good mechanical stability are promising to be obtained with the polymer blending technique^5^. Chitosan (CS) is an organic biodegradable polymer-chelating membrane with non-porous structure. A pure chitosan film shows low ionic conductivity while it is a desirable material for film formation^6^. In our earlier works, for the chitosan-based solid electrolytes, the reductions of silver ions to silver nanoparticles have been observed^7–11^. The existences of lone pair electrons on chitosan functional groups are found to be responsible for the reduction of silver ions to silver particles and complexation^12^. In our previous works we observed that chitosan incorporated with silver salts exhibits an almost amorphous phase^7,11^. On the other hand, polyethylene oxide (PEO) has been widely used as a good host macromolecule to prepare solid, gel and nanocomposite polymer electrolytes^13–16^. PEO-based SPEs have shown to exhibit a rich phase behavior, depending on the temperature, salt concentration, and thermal history. Moreover, high concentrations of a wide variety of salts are highly capable to be dissolved in PEO-based electrolytes^15,16^. PEO is a linear polymer with a helix structure. PEO is inexpensive and is amorphous above its melting point (T_m_). It is crystalline below 60 °C^17^. The semi-crystalline structure of PEO presents inherent problems as a polymer matrix for ion transport^18^. One of the most promising alternate choices of enhancing the amorphous phase and increase of ionic conductivity in PEO based electrolyte systems is blending of PEO with a suitable higher amorphous polymer^19,20^. Various polymers such as polyvinyl pyrrolidone (PVP)^19^, boroxine ring polymer (BP)^20^, polyacrylonitrile (PAN)^21,22^, and poly (dithiooxamide) (PDTOA)^23^ has been blended with PEO based electrolytes. The crystallinity of PEO based electrolytes extensively studied by various researchers through the XRD and DSC measurements^21,22,24,25^. Several authors reported the T_m_ value for PEO-based solid polymer electrolytes. Karmakar *et al*. reported the T_m_ of about 55.9 for PEO:LiI solid electrolyte system^24^. Chun-Guey *et al*.^21^, reported the T_m_ value of 56 for PEO:LiClO_4_ polymer electrolytes. The melting temperature (T_m_) is the temperature at which transformation of phase takes place in PEO based electrolytes and thus a steep increase of ionic conductivity may be observed from the plots of DC conductivity versus 1000/T. In former studies, a rapid increase of DC conductivity over 60 °C in PEO based solid polymer electrolytes has not been observed^23,26–29^. The finding of crystalline phases of PEO throughout the impedance plots has not been described previously^21–29^.

It was recognized that ion conducting electrolytes are the heart of electrochemical devices. Charge carriers accountable for transfer are cations of the dissolved salts. It is central for the materials to be first characterizes and after that chosen for a desired application based on their DC conductivity. It is well accepted in PEO that the alteration of phase transition happens from semi-crystalline to amorphous phase at temperatures higher than 60 °C^4^. Consequently, below the T_m_ of PEO extra semicircle due to crystalline phase of PEO may be appeared in impedance plots. The objective of this work is to show how the change of phase transition can be studied from the pattern of DC conductivity versus 1000/T. Furthermore, an obvious increase of crystalline phase as a consequence of an increase of various amounts of PEO to CS-based electrolyte has also predicted from the impedance plots. The morphological changes and structural appearances are helpful to understand the electrical properties. In the present work the role of phase transition of PEO on sudden increase of DC conductivity above 60 °C is presented in chitosan:PEO based blend electrolytes. To support the electrochemical impedance results and sudden increase in DC conductivity, various techniques, such as UV-vis, OM, SEM, XRD and DSC, were also used.

## Experimental Method

### Materials and sample preparation

In the present work, 80 wt.% of chitosan (≥75% deacetylated, average molecular weight 1.1 × 10^5^ g/mol) and 20 wt.% of silver nitrate (AgNO_3_) with a molecular weight 169.87 g/mol were used to prepare silver ion conducting polymer electrolyte using 1% acetic acid as a solvent. The chitosan (CS) powder was first dissolved in 100 ml of the acetic acid solvent. The solution was then stirred for more than 24 hours until a clear viscous solution of chitosan was obtained. The silver nitrate (20 wt.%) was then added to the prepared CS solutions with continuous stirring to prepare CS:AgNt electrolyte. On the other hand, different ratios of PEO were separately dissolved in acetonitrile with continuous stirring. Then, the PEO solutions were added to the prepared CS:AgNt solution to obtain polymer blend electrolytes. The polymer blend electrolyte samples were coded as CSPEN1, CSPEN2, CSPEN3, CSPEN4 and CSPEN5 for CS:AgNt incorporated with 10 wt.%, 20 wt.%, 30 wt.%, 40 wt.% and 50 wt.% of PEO, respectively. The mixtures were continuously stirred to obtain homogeneous solutions. Subsequently, the mixtures were cast in different Petri dishes and they were left to be dried to form films at room temperature. The films were transferred into a desiccator containing blue silica gel desiccant for further drying. This procedure produces solvent-free films.

### Characterization techniques

The ultraviolet-visible (UV-Vis) absorption spectra of the prepared films were recorded on a UV-vis spectrometer (V-570, Jasco, Japan) with the scanning range from 180 to 1000 nm. To investigate the surface microstructure of the nanocomposite films, optical micrograph (OM) images were taken. The images at adjusted magnification were taken by an optical microscope (MEIJI) through an attached camera-controlled (DINO-LITE) software. Furthermore, the surface morphology of the samples was examined by scanning electron micrograph (SEM) using the FEI Quanta 200 FESEM scanning electron microscope. X-Ray Diffraction XRD patterns were recorded using Empyrean X-ray diffractometer, (PANalytical, Netherland) with operating current and voltage of 40 mA and 40 kV, respectively. The samples were scanned with a beam of monochromatic CuKα X-radiation of wavelength (λ = 1.5406 Ǻ), and the glancing angles X-ray diffraction was in the range of 5° ≤ 2θ ≤ 80° with a step size of 0.1°.

The impedance of the films was measured using HIOKI 3531 Z Hi-tester within a frequency range of 50 Hz to 1000 kHz. The SPE blend films were cut into small discs (2 cm diameter) and sandwiched between two stainless steel electrodes under spring pressure. The measurements were also carried out at different temperatures ranging from 303 K to 363 K.

## Results and Discussion

### UV-vis analysis

**Figure 1:**
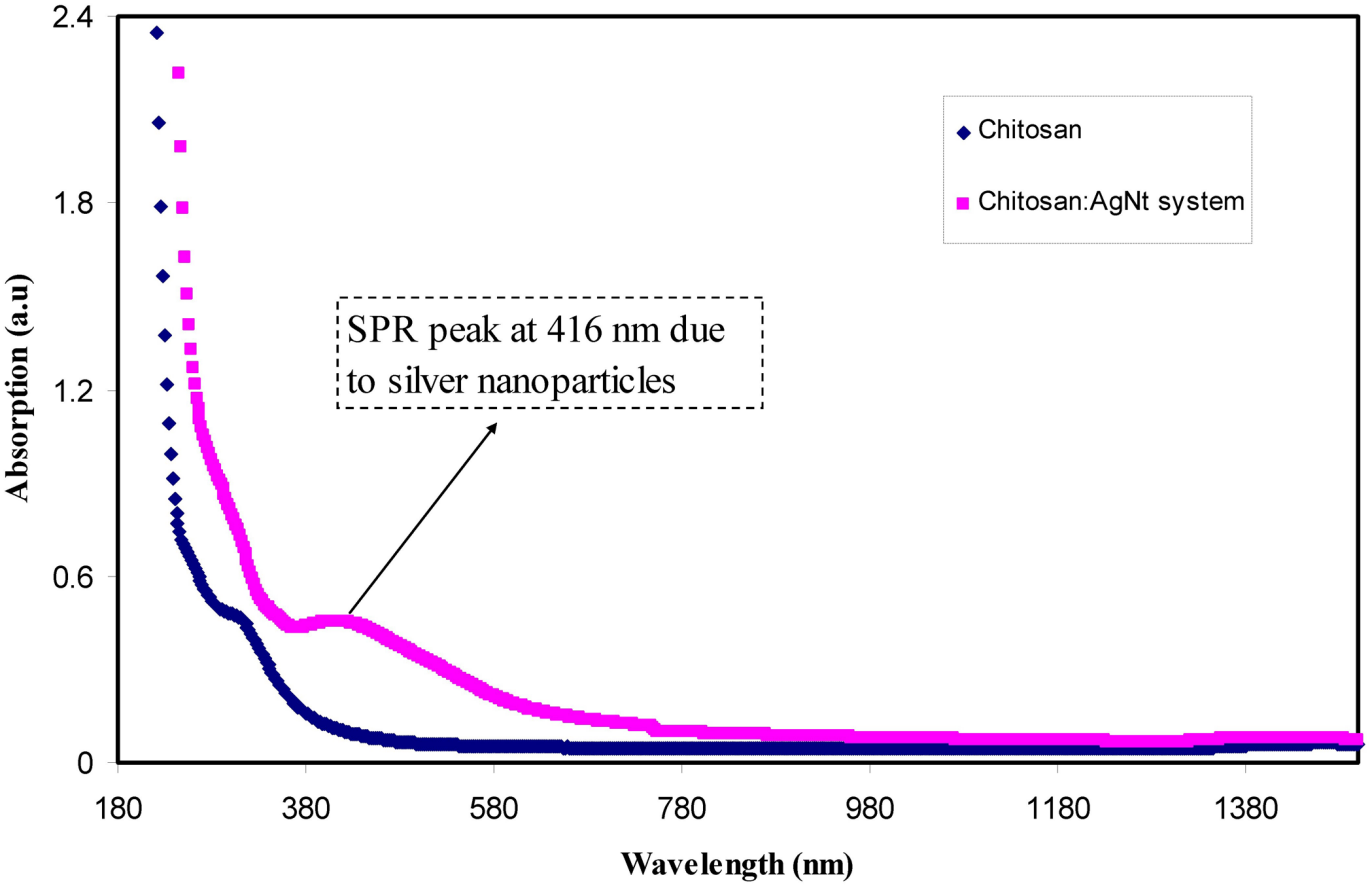
UV-vis spectra for pure CS and CS:AgNt samples.

**Figure 2:**
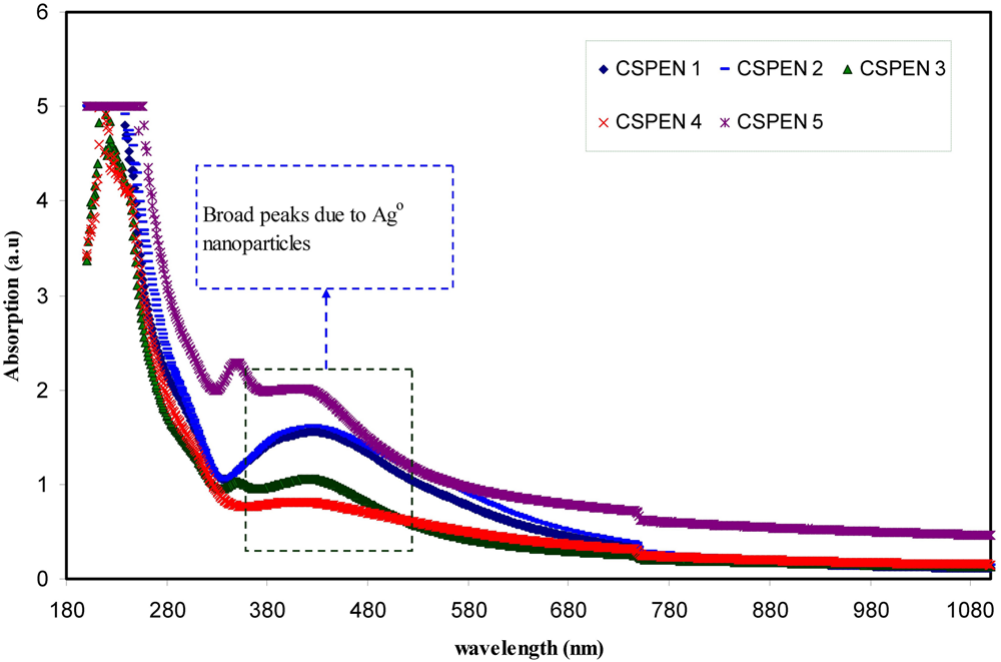
Uv-vis spectra for blended samples.

Figure 1 illustrates the absorption spectra for pure CS and CS:AgNt films. The broad absorption peak was observed at about 364 nm in the Uv-vis spectra of pure CS. This peak clearly can be seen in the Uv-vis spectrum of CSPE5 sample (see Fig. 2). The peak at 360 nm is a characteristic of π-π* transitions related to the presence of carbonyl groups (C=O) in CS polymer^7^. It is noticeable that no absorption peaks within the wavelength ranges of 380–580 nm are present for the pure CS, whereas a broad absorption peak at 416 nm were achieved for the CS:AgNt sample. This peak appeared again with improved intensity in the UV-vis spectra of the blended samples as shown in Fig. 2. Clearly the band appeared at 416 nm for CS:AgNt system (see Fig. 1) shifted in the blend samples and its intensity increased. As talked about in introduction section silver ions may reduce to silver nano-particles in CS based electrolytes. This can be considered as a big problem in front of the development of silver ion conducting based polymer electrolytes. That is the reduction of silver ions to metallic nano-particles can be regarded as the main drawbacks of silver ion conducting polymer electrolytes for electrochemical devise applications. Other researchers reported the reduction of silver ions in polymer electrolytes. Chandra *et al*.^30^, measured the contribution of electronic conductivity of metallic silver nanoparticles through the using of the Wagner’s polarization technique. They concluded that the electronic contribution to the conductivity increases as a result of the reduction of Ag^+^ ions into metallic silver particles in PEO-based electrolytes. UV-vis is a simple technique which can be used to detect the existence of silver nanoparticles in polymer-based electrolytes. It is well reported that particles with nanometer dimensions exhibit an intense absorption band so-called surface plasmon resonance (SPR) absorption band. This is originated from the movement of the conduction electrons within the particles, as a consequence of the electric field of incident light^31^. The local electromagnetic fields at the nanoparticle surface can be manipulated as well as enhanced through the plasmon resonance. Different metals exhibit different resonant photon wavelength^32^. The increase of band intensity (see Fig. 2) can be ascribed to the increase of silver nanoparticles^7,9–12^. The peak position, maximum absorbance and band shape of the surface plasmon resonance band are found to rely on particle structure, size, geometry, polydispersity and dielectric constant of the medium^31^. As it is well known, CS has two important functional groups, OH and NH groups^6^. Thus the results of UV-vis investigation reveal the formation of silver nanoparticles through the appearance of SPR peaks. Chitosan is a nontoxic natural biopolymer. An earlier study reported that the usages of environmentally-friendly materials in the generation of metal nanoparticles are of great importance^33^. Even the objective of this study is not to synthesize of metallic particles but the results show the availability of chitosan polymer to fabricate metallic silver particles. As stated in the introduction section ion conducting electrolytes are the central part of electrochemical devices and it is fundamental for the materials to be first analyzed and then chosen for a preferred application. In this study, to receive more information about the formation of silver nano-particles, morphological studies have been carried out.

### Morphological and Structural Study

**Figure 3:**
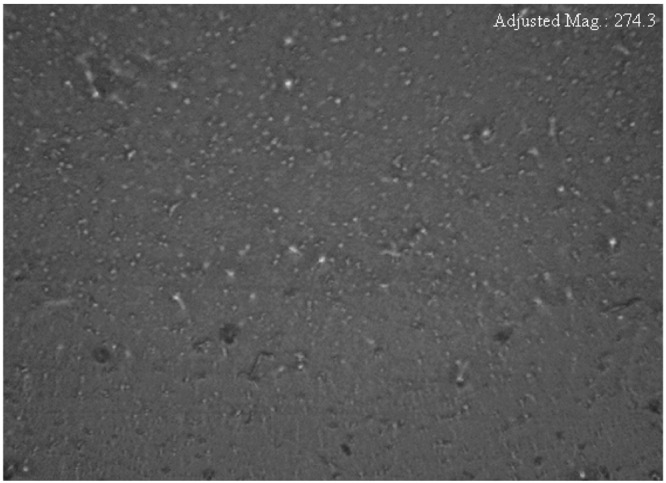
Optical micrograph image for CS:AgNt sample.

**Figure 4:**
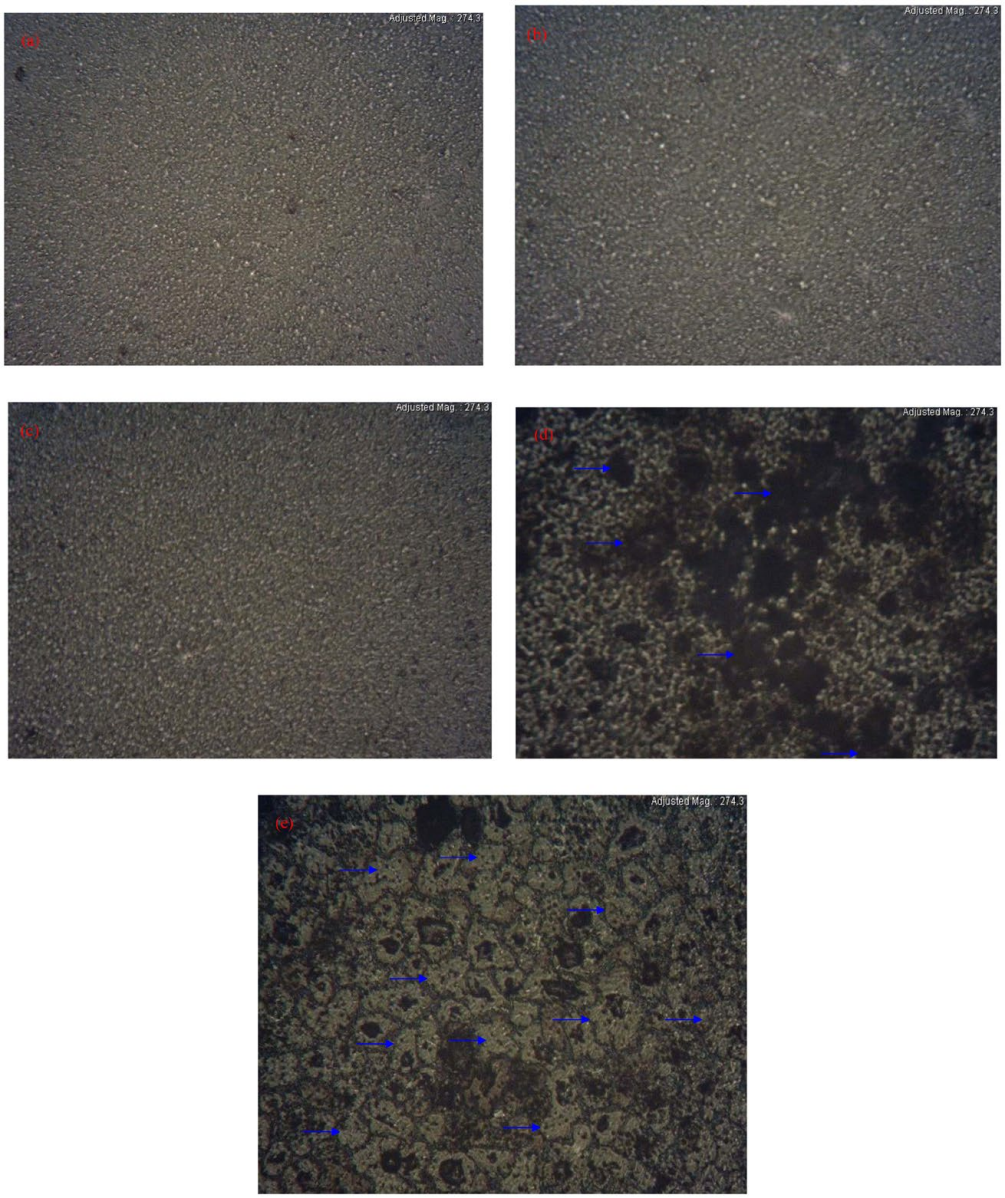
Optical micrograph image for (**a**) CSPEN1, (**b**) CSPEN2, (**c**) CSPEN3, (**d**) CSPEN4 (the dark regions indicated by green rows are amorphous phases) and (**e**) CSPEN5 (the spherulites indicated by green rows are distinguishable crystalline phases).

The surface morphology of CS:AgNt system which was observed by the optical microscopy (OM), is shown in Fig. 3. The tiny white spots can be seen on the surface of CS:AgNt film. These white spots were attributed to metallic silver nanoparticles. Thus the OM image in agreement with UV-vis result of CS:AgNt system (see Fig. 1). The surface morphology of the blended electrolyte samples were shown in Fig. 4(a–e). It is evident from the images that some white spots are appeared and their size increased as the PEO concentration was increased up to 30 wt.%. Similar evidence has also been observed using optical micrograph technique by other researchers^34^. These results are in consistent with the distinguishable SPR peaks of Fig. 2. It is apparent that the morphology of the samples are significantly distorted at the PEO concentration of 40 and 50 wt.%. From the prior studies, it is confirmed that the crystalline regions, which consists of a number of platelets or lamellae radiating from a nucleating centre (so- called spherulites), are emerged in the PEO based electrolytes^35–37^. The dark regions between the adjacent lamellae remain amorphous^37^. Previous studies confirmed that spherulite is interrelated to crystalline region and the region between spherulite interface ascribed to amorphous phase^24,35–37^. Crystallization and melting phenomena are identified to have a noteworthy impact on the electrical properties of semicrystalline polymer electrolytes. Increasing stiffness and alignment of polymer chains into lamellae powerfully affect the ionic conductivity, through the blocking of ions and reduced polymer chain motions^36^. It is well recognized that the degree of crystallinity of the polymer electrolyte has an important role on the ionic conductivity^35^. Reddy and Chu have studied the morphology of PEO: potassium iodide (KI) electrolyte using the optical micrograph method^38^. They observed that the size of the spherulites decreases with increasing the KI concentration and thus increasing the dark region. In this study, the amount of dissolved salt is fixed through the host chitosan polymer. Therefore, the increase of spherulites is predictable here since the concentration of PEO is increased, while the CS:AgNt system is kept constant. From Fig. 4d, it is obvious that the crystalline enlargement can be evidently seen at 40 wt.% PEO. The manifestation of interconnected spherulitic structure with some amorphous region (see Fig. 4e) is evidence for the fact that incorporation of a high amount of semi-crystalline PEO into the CS:AgNt system can affect the crystalline structure of the system, which may reduce the ionic conductivity. Marzantowicz at al, have studied the morphology and impedance spectroscopy of PEO based electrolytes^35^. They observed that the diameter of the second semicircle directly increased with increasing the crystallization of PEO based electrolyte. Consequently, the examination of impedance spectra may provide more insights on the crystallization phenomena in the present work.

**Figure 5:**
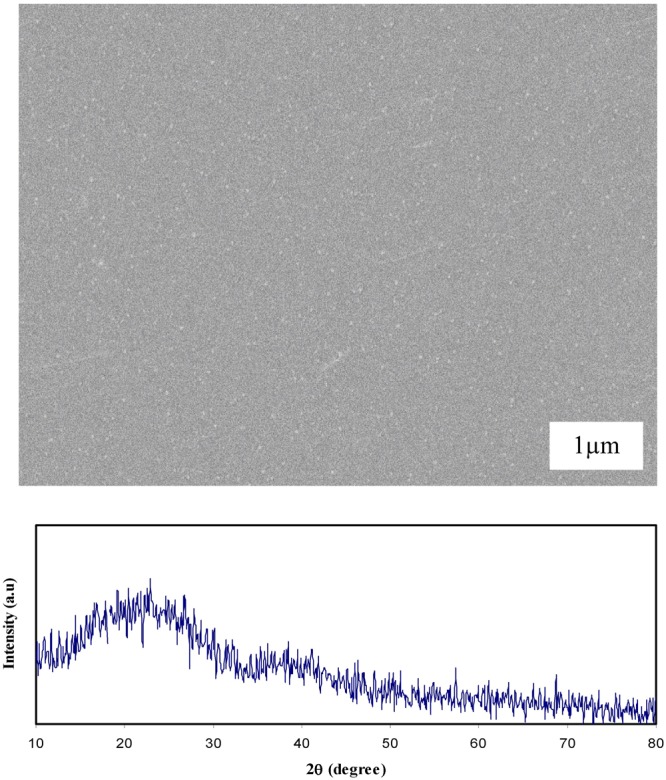
SEM image and XRD result for CS:AgNt system.

**Figure 6:**
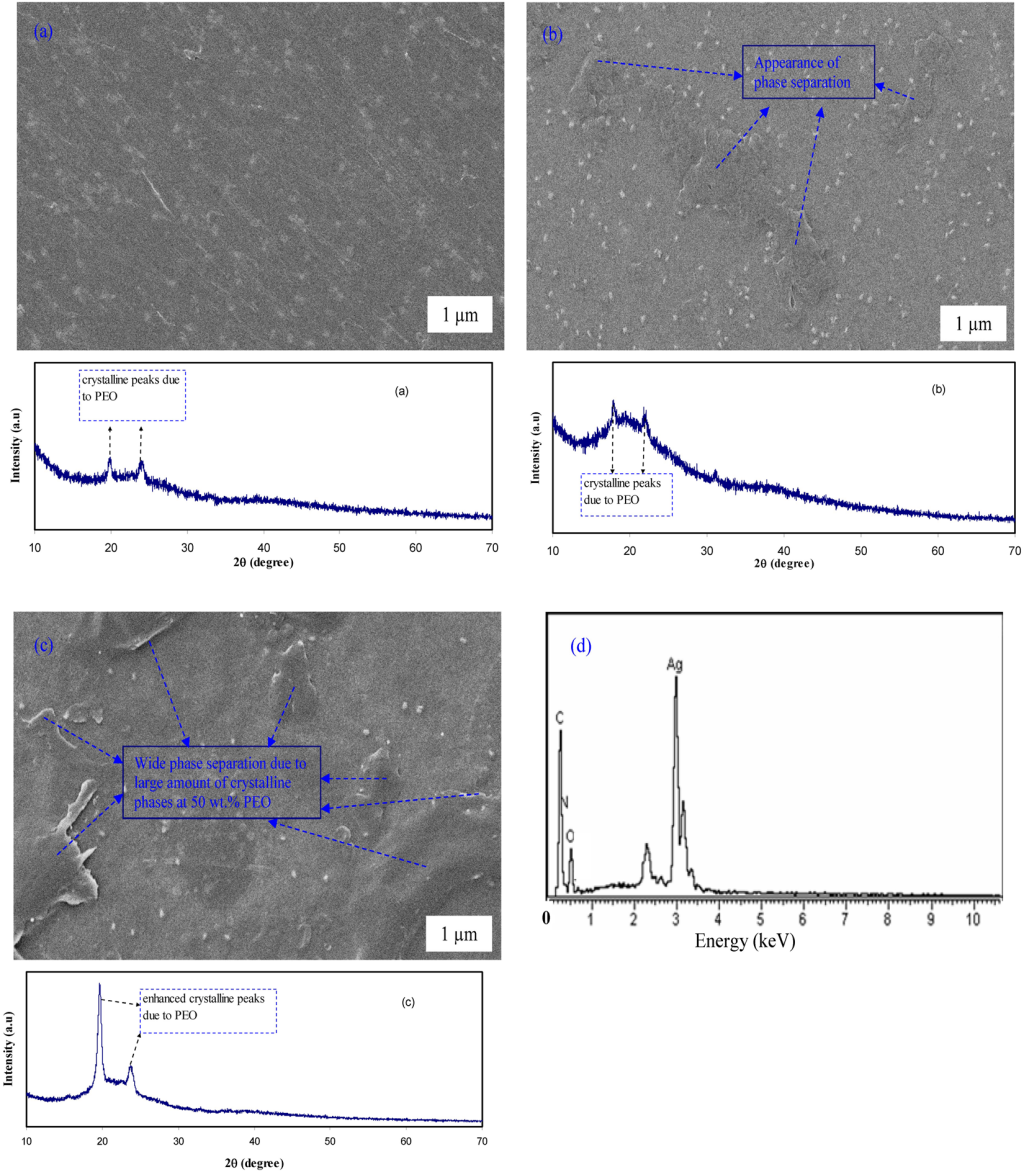
SEM images and XRD results for (**a**) CSPEN1, (**b**) CSPEN3, (**c**) CSPEN5 samples and (**d**) EDX analysis for white specs appeared on CSPEN3 samples.

The SEM images have been taken for the selected samples to support the UV-vis and OM results. The SEM technique was found to be an efficient way to study the structure and morphology of the surface of the samples. On the other hand, one of SEM technique’s advantages is that the choice of magnification is broadly allowing us to simply focus on a particular area of the sample^39^. It is well-known that the surface morphology and structure of the polymer electrolyte films are the key properties to identify their behavior. The electron image was taken at 1000 × magnification. Prior to examination, the films were attached to aluminum holder using a conductive tape and then coated with a thin layer of gold. The SEM image and XRD result for CS:AgNt (base material) system is shown in Fig. 5. Small white specs due to Ag° nanoparticles appear on the film surface. The non-existent of phase separation and broad XRD peaks reveal the amorphous structure of CS:AgNt sample. The SEM images for selected polymer blend electrolyte films are shown in Fig. 6(a–c). The SEM characterizations were carried out in order to observe the morphological changes in the blended samples. As can be seen from the figure, the film with 10 wt.% of PEO reveals a smooth morphology with some white specs, indicating the formation of silver nanoparticles. The EDX spectrum was taken on white spots for CSPEN3 samples (Fig. 6b). Clear peaks for metallic silver particles could be seen in the EDX spectrum (see Fig. 6d). Therefore, the SPR peaks appeared in Figs 1 and 2 for all the samples are strongly supported by the obtained SEM and EDX results. It is also important to state that, in our previous works, white spots and chains of silver nano-particles was obtained in our SEM image analysis carried out on chitosan samples incorporated with silver salts^7,8,10,11,40^. An irregular wavelike surface structure appeared in Fig. 6a is a result of the polymer-salt complexation. It is well reported that smooth morphology appearance is closely associated with the nature of amorphous phase of the polymer blend electrolyte complex^41^. The phase separation was found to be little at 10 wt.% of PEO (see Fig. 6a), while an obvious phase separation was observed at 30 and huge amount of phase separation can be seen at 50 wt.% of PEO as depicted in Fig. 6b and Fig. 6c. Clearly, the wide phase separation regions at 50 wt.% PEO were occurred. It is well reported that the absence of phase separation and appearance of smooth surface morphology in blend electrolytes are evidences for the amorphous nature of the system^41^. The XRD results were provided along the morphological appearances in Fig. 6a–c. The two obvious peaks appeared at 2θ ranges from 17° to 23° are ascribed to crystalline peaks of PEO as reported in literature^16,19,22,24,25^. Noticeably with increasing PEO concentration the strength of crystalline peaks owing to PEO increased and the amorphous phases are suppressed. Thus the XRD results seriously support the OM images and SEM micrographs. In our previous work, we have used SEM technique to detect crystalline structures attributing to the ion pairs formation in chitosan:NaTf solid electrolyte system at high NaTf salt concentration. The SEM results were utilized to explain the drop in DC conductivity in chitosan:NaTf system at high NaTf salt concentration^39^. Kadir *et al*., have also used SEM to identify the protruded crystalline structures of salts in chitosan based solid electrolytes at high salt concentrations^42,43^. The results of the present work illustrate that SEM method can also be used to detect the phase separation in blend polymer electrolytes. The appearance of huge amount of phase separation is an evidence for the large amount of crystalline phases as found in XRD results. The results of OM study strongly supported the SEM images.

### Impedance and Bode Plot Study

**Figure 7:**
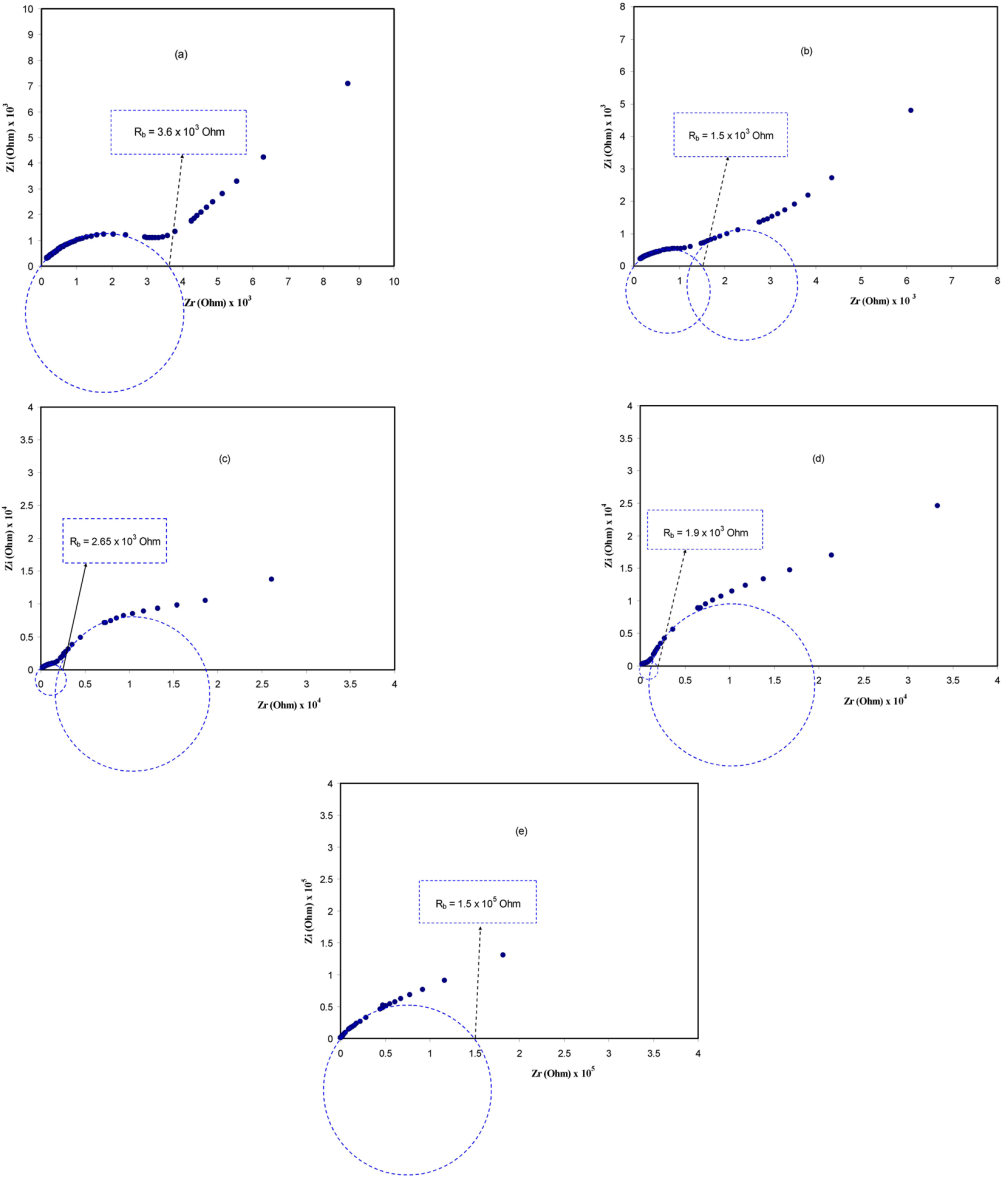
Impedance plots for (**a**) CSPEN1, (**b**) CSPEN2, (**c**) CSPEN3, (**d**) CSPEN4 and (**e**) CSPEN5 films at ambient temperature.

Figure 7(a–e) demonstrates the impedance spectra for all the polymer blend electrolyte samples incorporated with different concentration of PEO at room temperature (303 K). Typical ac impedance plot usually exhibits two distinct regions: The high frequency semi-circle and the low-frequency spike regions. The semi-circle and spike observed at high and low frequency regions have been ascribed to the process of ionic conduction through the bulk of polymer electrolytes and the effect of blocking electrodes (electrode polarization), respectively^44^. The electrolyte/electrode interface can be thought as a single capacitor, since the blocking electrodes have been employed in the impedance analysis. In the impedance plot, the capacitor should exhibit a vertical spike when it is considered to be ideal^45,46^. It is obvious that the second semicircle is observed at 20 wt.% PEO. It is attractive here to note that the diameter of the second semicircle has been increased with increasing PEO concentration up to 40 wt.% and the first semicircle shifted towards the origin. Eventually, when the PEO concentration reaches 50 wt.%, the first semicircle disappeared and the resistivity of the sample considerably increased. In other words at 30 wt.%, 40wt.% and 50 wt.% of PEO more crystalline fractions due to PEO was introduced to the CS:AgNt (base material) system, consequently the second semicircle suppresses the first semicircle and thus difficult to distinguish it. In this work we concluded the fact that: The diameter of second semicircle in impedance plots has found to be increasing with increasing PEO concentration, i. e., the second semicircle is related to the crystalline fraction. At 50 wt.% of PEO only one semicircle with big diameter can be distinguished, which indicated the increase of resistivity. It is well documented in polymer electrolytes the crystalline regions block the ionic motion and thus a decrease in conductivity. These results are in accordance with our morphological appearance as seen in Figs 4 and 6. Previous study also reported that development of spherulite primarily has a moderately minute effect on the conductivity however the conductivity very much decreases as the crystallites begun to comprise a bulky fraction of the polymer^35^. The most probable explanation for the decrease in conductivity is the densification of the structure (i.e., the increase of crystalline arrangement between existing lamellae). Consequently, this causes the charge carrier transport to be hindered in the remaining amorphous phase. In such a compact structure, a small modification of amorphous phase content can produce an interruption in continuity of the easy conduction path. On the other hand, the sample stiffness and a loss of contact with electrodes can be another reason for such large drop of conductivity^36^. Many studies have been established that the process of ion transport in amorphous portion of polymer electrolytes is high and its mobility decreases with increasing crystalline regions^45,47,48^. The results achieved in the present work reveal that the properties and structure of materials are strongly correlated. Comparing the graphs of Figs 6 and 7, it is easy to grasp that the growth of crystalline region in polymer electrolytes is an obstacle in front of researchers to develop a solid electrolyte system with high DC conductivity at room temperature. From the OM images, it is appeared that the crystalline region increases with increasing the PEO concentration and consequently the second semicircle increases continuously until disappearing the first semicircle and thus a drop in DC conductivity occurs. A clear phase separation (SEM images) and intense crystalline peaks (XRD patterns) observed in Fig. 6 strongly supports the fact that a wide region of crystalline phase has developed at high PEO concentration. Consequently, a drop in conductivity can be predictable because ion transport almost occurs in an amorphous phase of polymer electrolytes. The role of crystalline and amorphous phases on conductivity could be more understood from the study of impedance spectra at selected temperatures as can be seen in later sections.

**Figure 8:**
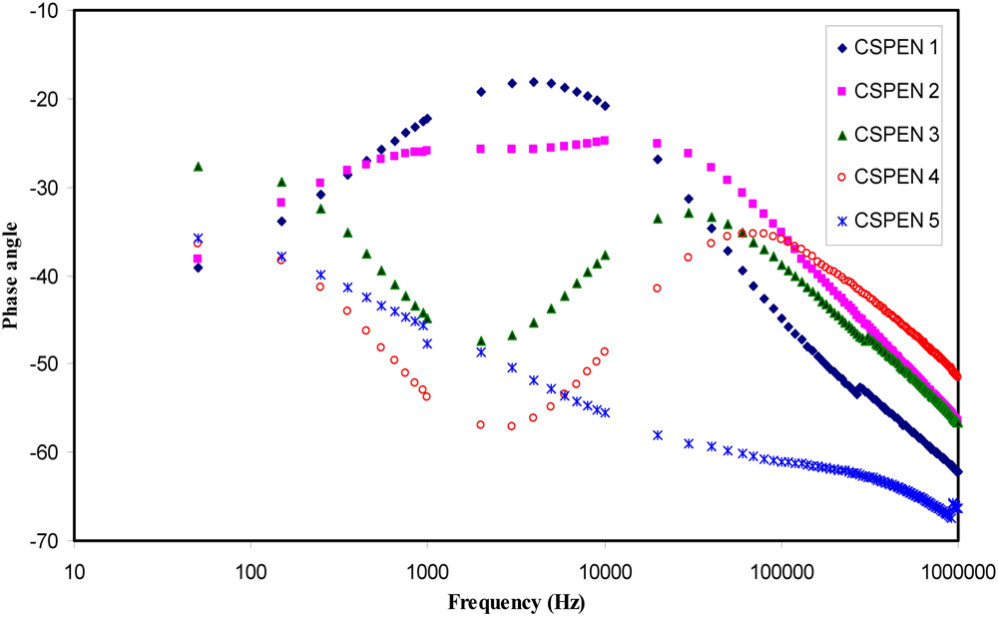
Bode plot (phase angle vs frequency) for all the blend electrolyte samples.

**Figure 9:**
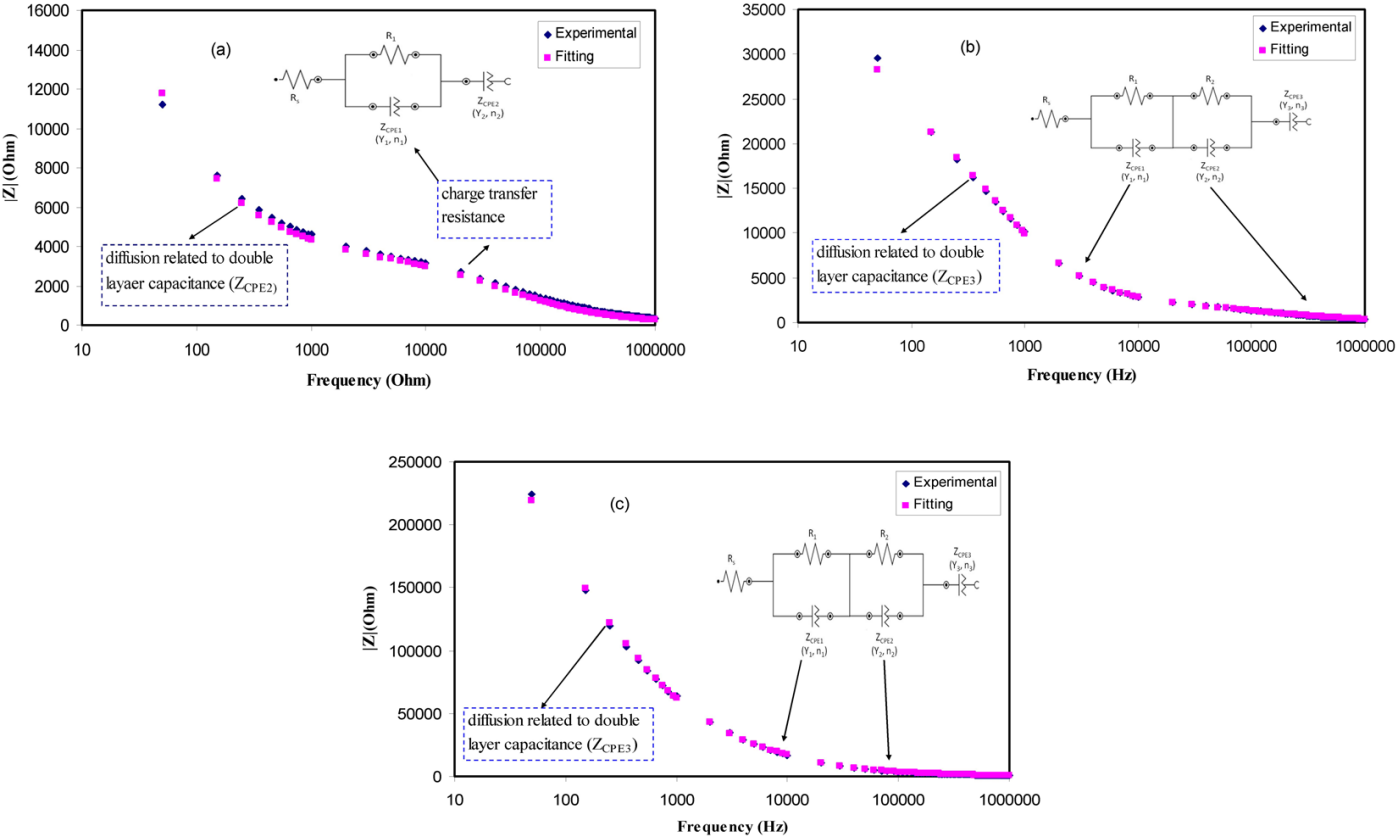
Bode magnitudes (experimental and fitting) for (**a**) CSPEN1, (**b**) CSPEN2 and CSPEN5 blend electrolyte samples.

To support our interpretation and our proposed semicircles for Nyquist plots we have studied the Bode plots. From the electrochemical viewpoints Bode plots are crucial to be studied in order to understand the charge transfer in electrolyte materials. Figure 8 shows the Bode plot (phase angle vs frequency) for the all blend electrolyte samples. It is obvious that the at 10 wt. of PEO one peak can be distinguished while at higher PEO concentration two peaks can be separated as observed in impedance plots. According to Ali Eftekhari^49^, three regions should be distinguished in Bode plots which are capacitive, diffusion and charge transfer regions. The capacitive region (plateau region) can be manifested at a very low frequency usually from 10^−2^ to 10° Hz. Thus due to frequency limitations this region cannot be evident in the Bode plot of the present work. As discussed in impedance plots (see Fig. 7), the first peak is related to ion transfer in amorphous phase of the electrolytes and the second peak is ascribed to crystalline phase of PEO and its strength increases until suppress the first peak at 50 wt.% of PEO. Thus the increase of PEO up to 50 wt.% responsible for the increase of resistivity. To establish the fact that each peak corresponds to an electric circuit, Bode magnitude was plotted for selected samples as depicted in Fig. 9(a–c). The fitting data corresponds to electrical equivalent circuit of each sample. The insets of Fig. 9 show the equivalent circuits. It is clear from Fig. 7a that the impedance of the system consists of one semicircle and spike at low frequency region. Thus we have one equivalent circuit for this system. For other impedance plots of Fig. 7 two semicircles can be drawn. Each semicircle corresponds to an equivalent electric circuit. Consequently, Bode magnitude strongly supports the Bode plot of Fig. 8 and both of them confirms our interpretation and our proposed semicircles for the impedance plots (see Fig. 7). That is, each semicircle represents the parallel combination of a resistor and capacitor. The spikes at low frequency regions are usually represented by constant phase elements (CPE) which are in series with equivalent circuit elements. Other researchers used equivalent circuits to interpret the Bode and impedance plots^50,51^.

### DC Conductivity Analysis

**Figure 10:**
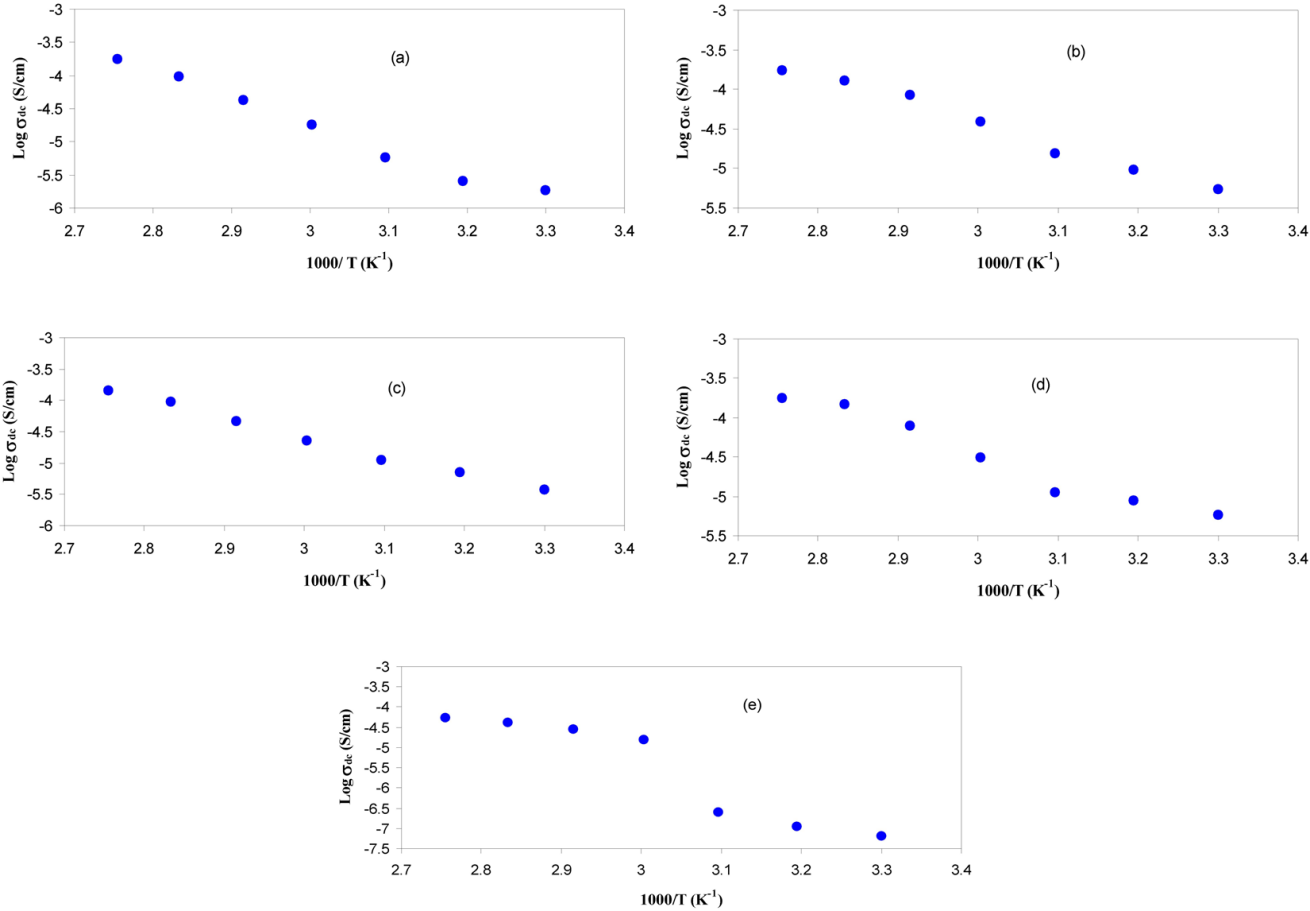
DC conductivity versus 1000/T for (**a**) CSPEN1, (**b**) CSPEN2, (**c**) CSPEN3, (**d**) CSPEN4 and (**e**) CSPEN5 films.

**Figure 11:**
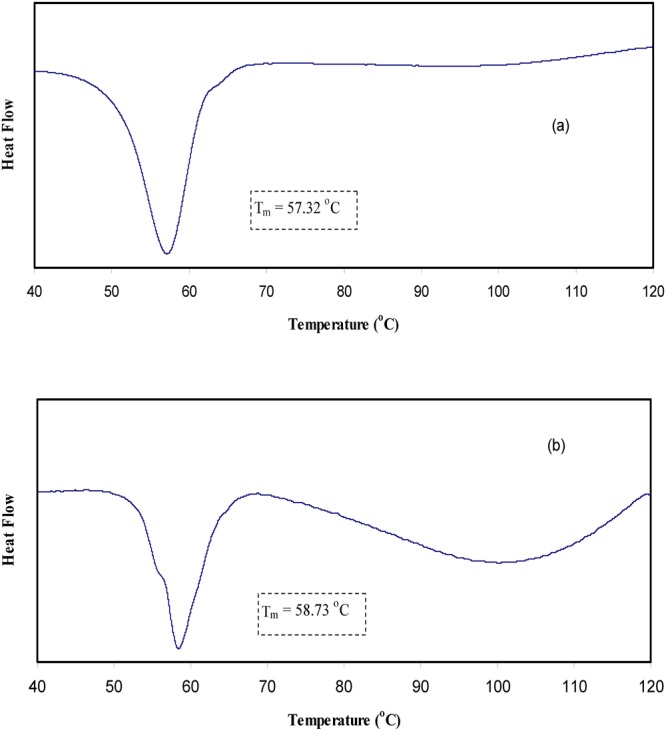
DSC measurements for (**a**) CSPE3 blend sample and (**b**) CSPE5 blend sample.

**Figure 12:**
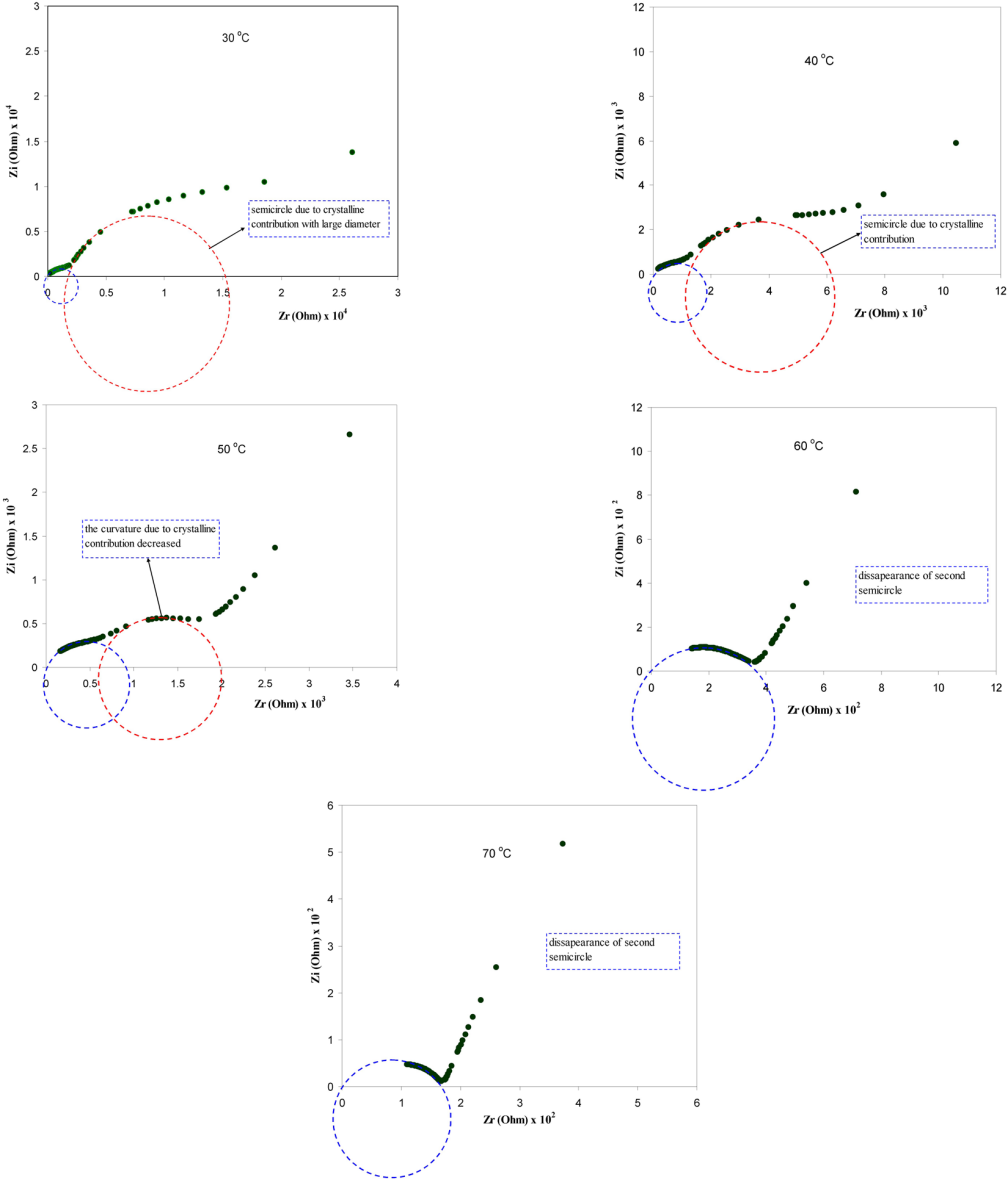
Impedance plots for CSPEN3 sample at different temperatures. Clearly from 30 to 50 °C the diameter of second semicircle in impedance plots decreases. From 60 °C and above only one semicircle can be distinguished.

**Figure 13:**
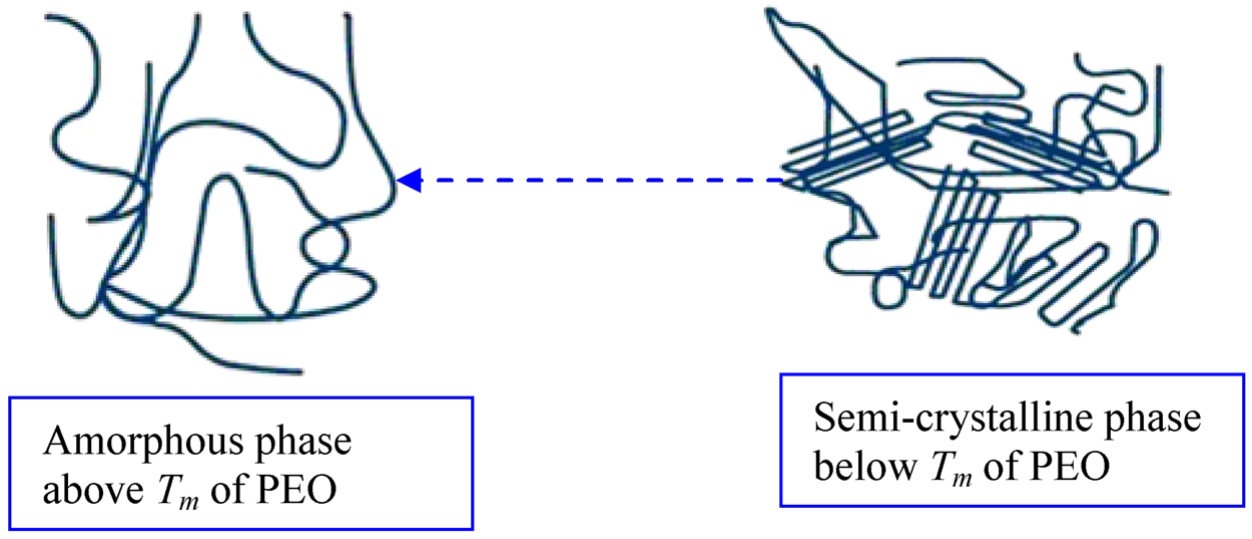
Shows the proposed phase of change from semi-crystalline to amorphous phase above *T*_*m*_ of PEO.

Figure 10 shows the DC conductivity versus 1000/T for the blend electrolyte samples. Two regions can be distinct throughout the temperature range of interest. At low temperatures, the DC conductivity gradually increases with increasing temperature, while above 333 K an abrupt change in DC conductivity is observed. This rapid increase of DC conductivity at high temperatures can be attributed to the phase change of PEO from semi-crystalline to amorphous phase. Earlier studies have also correlated the abrupt increases of DC conductivity at around 60 °C to the typical semi-crystalline amorphous phase transition in PEO^4,52^. The linear variation of DC conductivity with temperature below 60 °C implies that ion transport occurs through the thermally activated processes and thus obeys an Arrhenius behavior^52^. Clearly, the temperature dependence of DC conductivity shows curvature above 60 °C. The expansion of polymer matrix with temperature increase forms free volume and empty spaces, in which the ions migrate. This facilitates the ion mobility and reduces the ion cloud effect at electrode/electrolyte interface^2^. It is established that greater ionic diffusivity can be accomplished in the case of amorphous polymers^53^. As amorphous zones gradually develop in the region II (above 60 °C), the polymer chain obtains faster internal modes, in which segmental motion is produced due to the bond rotations. This consecutively favors the inter-chain hopping movement of ions, and thus leads to an improvement of conductivity for the polymer electrolyte. Above the melting point, the order of variation in the conductivity is small due to the complete amorphous nature of the electrolyte samples^54^. The curvatures of the Arrhenius-type plots show that the ionic conduction follows the relation of Vogel–Tamman–Fulcher (VTF)^8^, in which the transport properties in a viscous matrix are described. The VTF relation supports the idea of the existence of ion movement through the plasticizer-rich phase, i.e^55^.

1 $${\sigma }_{dc}=A{T}^{-1/2}{\exp }^{(\frac{-{E}_{a}}{{K}_{B}(T-{T}_{O})})}$$

where, *E*_*a*_ and A are fitting constants that describe the activation energy and the number of charge carriers, respectively, *K*_*B*_ is the Boltzmann constant and *T*_*o*_ refers to the system at equilibrium related to zero configurational entropy. It is found that *T*_*o*_ is approximately equal to *T*_*g*_ − 50 K, where *T*_*g*_ refers to the thermodynamic glass transition temperature of the system^54,55^. Therefore, the segmental motion either creates a channel for the ions to move or allows the ions to hop from one site to another. In other words, the translational ionic motion is facilitated by the segmental movement of the polymer^45,56^. To verify that the abrupt increase of DC conductivity is associated to the alteration of phase of PEO from crystalline to amorphous phase above 60 °C the DSC was carried out on selected samples. Figure 11 shows the DSC plots for the CSPE3 and CSPE 5 films. Clear peaks due to the melting point of PEO were observed at 57.32 °C and 58.73 °C for CSPE3 and CSPE 5 samples, respectively. The results of DSC measurement are analogous with those reported in the literature for PEO based blend and composite electrolytes^15,23–25^. More insights into the happening of change of phase in PEO based blend electrolytes of the present work may be accomplished throughout the impedance study at a different temperature. We showed in section 3.3 that the growth of second semicircle in impedance plots is associated to the crystalline phase of PEO content. Based on temperature dependent DC conductivity and DSC measurements the second semicircle which is due to crystalline phase must be disappeared at 60 °C and above in impedance spectra. Figure 12 shows the impedance plots of CSPN3 system at various temperatures. It is evident from the figure that the diameter of the second semi-circle is decreased with increasing temperature from 40 °C to 50 °C. The second peak resulting from crystalline region was disappeared at 60 °C and 70 °C. It is important to note that, the existence of the slanted points and incomplete semi-circle at low and high frequencies, respectively, are evidence for the amorphous nature of the samples at high temperatures. In addition to our observation, other researchers have also related the observed spike and incomplete semicircle to the bulk conductivity of polymer electrolytes and polymer nanocomposites, respectively^39,42,48,57–59^. This indicates that the change of phase transition from crystalline to amorphous occurs above 60 °C and it has a great role on ion transport. Therefore, the abrupt increase of DC conductivity at ~60 °C is a resultant of amorphousness of the samples at high temperatures. The schematic diagram shown in Fig. 13 illustrates the transition from semi-crystalline to amorphous phase with increasing temperature to above the T_m_ of PEO polymer. Comparing Figs 7 and 12 is vital to understand the increase and decrease of crystalline phases. In Fig. 7 it was observed that with increasing PEO ratio the second semicircle diameter also increased due to the increase of crystalline phase. However in Fig. 12 it was shown that with increasing temperature the second semicircle size in impedance plots decreased and from 60 °C and above only one semicircle can be distinguished which indicated the homogeneity of the system (i. e., completely amorphous phase). Other researchers were also observed incomplete semicircular arcs in the high frequency region and spikes at low frequency regions for high ion conducting membranes based on chitosan^60,61^. It is well established that polymer electrolytes are heterogeneous materials, owing to the existing of both amorphous and crystalline phases. Thus with increasing temperature to T_m_ of PEO the system was changed to homogeneous material with high DC conductivity. The results of the present work are an example of precision in measurement. Exactly at about of T_m_ of PEO the second semicircle disappeared.

## Conclusions

Various amounts of PEO were incorporated into the CS:AgNt system to fabricate blend polymer electrolytes with enhanced ion transport mechanism. From the UV-vis spectra measurements, wide surface plasmonic resonance (SPR) absorption peaks owing to metallic silver particles were observed. The result was further supported by optical microscopy (OM), SEM and EDX analysis. Small white specs were found on the surface of the samples using the both imaging (OM and SEM) techniques. The spherulites observed in OM micrographs and phase divisions emerged in SEM images are ascribed to crystalline phases of PEO polymer. The manifestation of powerful crystalline peaks of PEO at 50 wt.% is answerable for observing numerous spherulites in OM images and wide phase separations in SEM micrographs. The strong crystalline peaks emerged at 50 wt.% of PEO is responsible for the raise of resistivity. The dominant of crystalline phase at 50 wt.% PEO is accountable for vanishing the first semicircle in impedance plots at room temperature. The DC conductivity was determined to increase with temperature slowly at low temperature ranges, while an rapid change in DC conductivity was observed above 333 K. The rapid increase of DC conductivity at high temperatures is correlated to the change of phase transition of PEO from semi-crystalline to amorphous phase as indicated from DSC measurements. The results of impedance study for various concentrations of PEO at ambient temperature and impedance spectra at selected temperatures reveals that impedance measurement can be used as a unique approach to exploring the microstructure of polymer electrolyte based materials.
